# Living with vulval lichen sclerosus: a qualitative interview study*

**DOI:** 10.1111/bjd.21777

**Published:** 2022-08-22

**Authors:** Susanne Arnold, Sheryl Fernando, Sophie Rees

**Affiliations:** ^1^ Warwick Clinical Trials Unit University of Warwick Gibbet Hill Road Coventry UK; ^2^ Patient Advisory Panel Member; ^3^ Bristol Trials Centre, Bristol Medical School University of Bristol Bristol UK

## Abstract

**Background:**

Vulval lichen sclerosus (VLS) is a chronic inflammatory condition predominantly affecting the anogenital skin. Symptoms can be distressing and affect quality of life and everyday activities. Very little research has been undertaken to explore the experience of living with VLS from the perspective of people with the condition.

**Objectives:**

To understand individuals’ experiences of VLS and its impact on their lives.

**Participants and methods:**

Semi‐structured remote (telephone or video) interviews were conducted with a purposive sample of 20 women living in the UK with VLS recruited via online support groups and social media. Data collection and analysis was informed by social constructionist grounded theory, using a constant comparison method.

**Results:**

We developed three themes to interpret the experience of living with VLS: missed opportunities (participants experienced delayed diagnosis, lack of information and disempowering encounters with healthcare professionals); learning to live with a long‐term condition (the amount of work involved in learning how to self‐manage the disease and the impact on everyday life); a secret life (experiences of the condition were often shrouded in secrecy, and there was stigma associated with a vulval skin condition resulting in them feeling isolated and lonely).

**Conclusions:**

Patients attending healthcare appointments with vulval complaints should be examined and LS should be considered as a diagnosis. Healthcare professionals’ awareness and knowledge of VLS needs to be improved and they should avoid language which is blaming or minimizing of patients’ experiences. VLS is a chronic condition and patients need to be supported in self‐management. Support groups may be a source of support and information but can also be challenging when hearing others’ difficult experiences. Wider public health educational activities are needed to change societal attitudes towards female genitals and tackle the stigma around vulval conditions.

**What is already known about this topic?**

- Vulval lichen sclerosus (VLS) can have a profound impact on quality of life and self‐identity but is relatively underexplored from the perspective of those living with the condition.

**What does this study add?**

- In‐depth findings about the experiences of living with VLS including ongoing issues with timely diagnosis, learning to live with a long‐term condition and the secrecy and stigma about the condition.
- The needs of women with symptoms of and diagnoses of VLS are not being met sufficiently by the healthcare system.

**What are the clinical implications of this work?**

- Healthcare professionals should consider addressing knowledge gaps in vulval conditions including VLS to prevent delayed diagnosis and avoid the use of certain terminology which can minimize patients’ experiences.
- Patients with vulval complaints should be examined and LS should be considered as a diagnosis.
- Regular follow‐up would reflect its chronic nature and could provide patients with reassurance and confidence in self‐management.
- Wider public health activities are needed to change societal attitudes and tackle stigma around vulval conditions.

## Graphical abstract

Vulval lichen sclerosus can have a profound impact on quality of life and self‐identity but is relatively under‐explored from the perspective of those living with the condition. Using qualitative methods, the aim of this study was to explore individuals’ experiences of vulval lichen sclerosus and its impact on their lives. We developed three themes to interpret the experience of living with vulval lichen sclerosus: missed opportunities, learning to live with a long‐term condition, and a secret life.

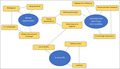

**Linked Comment:** V. Sivalingam and K. Tamber. Br J Dermatol 2022; 187:840.

Plain language summary available online

Vulval lichen sclerosus (VLS) is a chronic inflammatory condition typically affecting the anogenital area. Symptoms include intense itching, burning, formation of white lesions, small painful tears, and architectural changes such as burying of the clitoris and labial fusion, sometimes requiring reconstructive gynaecological surgery.^1^, ^2^ Women with VLS are also at an increased risk of developing vulval cancer.^3^

The incidence of VLS remains unclear and is likely to be underestimated, although it has been suggested that prevalence in adult females is up to 3%, presenting most commonly in prepubescent and postmenopausal women.^4^ Diagnosis is based on clinical examination but may be confirmed by biopsy.^2^ However, it can take many years for women to be diagnosed. Symptomatic individuals may not seek help due to embarrassment, or when they do, doctors may not perform an examination, or may not recognize VLS when they do.^3^, ^5^

There is no cure for VLS, but recommended treatment is an ultrapotent steroid ointment.^2^ Rates of symptom reduction are reported as between 54% and 96% of women, although ≈ 80% experience recurrence within 4 years.^6^ The long‐term impact of steroid ointment is currently unclear,^7^ although some evidence suggests it is safe and prevents cancer.^8^

The condition can profoundly impact quality of life and self‐identity but is relatively underexplored from the perspective of those with VLS.^9^ It can affect functional activities such as toileting, sitting or walking, and sexual activity, as well as self‐esteem and confidence.^10^ A 2015 survey found that one in five women with a vulval condition had considered suicide or self‐harm as a result of the condition.^11^

Most existing research on VLS and quality of life has used quantitative methods^12^ focusing on sexual dysfunction.^10^, ^13^, ^14^, ^15^, ^16^ In a 2019 systematic review exploring women’s lived experiences of VLS,^9^ we found three publications for inclusion: an analysis of online blogs/forum entries^17^ and two reporting findings from a Dutch study of 19 women’s experiences of vulval surgery to relieve dyspareunia.^18^, ^19^

In 2021, a thematic analysis^20^ of 202 online posts from two open forums explored what issues women with VLS choose to discuss. Five themes emerged: problems with obtaining a diagnosis; rationalization and validation of women’s experience; self‐management of the condition; seeking advice and guidance; and the social repercussions of VLS. Other research has involved women with vulval dermatoses including VLS.^21^ Most recently, in the interview study by Sadownik *et al*.^22^ (including seven women with VLS), a global theme of suffering, incorporating grieving, isolation and interference, was developed. This encompassed several basic themes including silence, lack of validation and support, and feeling different.^22^

In a survey study^23^ exploring quality of life of women with vulval and nonvulval inflammatory dermatoses (64% with VLS), participants were asked to describe challenges they experienced when seeking a diagnosis. Four themes were developed: provider‐based challenges including misattribution of symptoms and insensitive communication; system‐based challenges incorporating provider education; personal challenges including reduced healthcare efficacy and embarrassment; and positive and negative emotional responses and impact.^23^

VLS remains an under‐researched disorder. Further evidence is badly needed to identify how to best help women, ensuring their voices are heard. In a 2018 James Lind Alliance Priority Setting Partnership, the question ‘What is the impact on quality of life?’ was prioritized in the top 10 research priorities by patients, clinicians and researchers.^24^ The aim of this study was to understand women’s experiences of VLS and its impact on their lives from their own perspectives.

## Participants and methods

### Study design

This was a qualitative semi‐structured interview study with a purposive sample of women living with VLS from across the UK.

### Sampling and recruitment

Participants were ≥ 18 years old, reported a diagnosis of VLS (either by clinical examination or biopsy) and were recruited via social networks (online VLS support groups, social media, mailing lists) and poster advertisements (to reach those without access to online resources). Recruitment materials were developed alongside our patient advisory panel. We recognize that those who do not identify as women may also experience VLS, and we ensured our recruitment materials were gender‐neutral and advertised through a prominent trans research partnership.

Individuals who expressed an interest were sent a participant information leaflet and given the opportunity to ask further questions. Those willing to participate provided demographic data. This was used in purposive sampling to identify and gain perspectives from people of different ages, ethnicities and with different duration of symptoms. The final sample size was determined by ‘meaning saturation’, which is achieved by collecting rich data and is not simply about seeing patterns repeating in the data, but about how well fleshed out the categories in the evolving analysis are.^25^ We anticipated that 20–25 interviews would provide sufficient data to enable an interpretation of the experiences.

### Data collection

Data was collected during the COVID‐19 pandemic. Therefore, all interviews were undertaken by telephone or virtual video call using Microsoft Teams. Interviews were audio‐recorded. Verbal consent was audio‐recorded prior to the interview.

All interviews were conducted by S.A. and S.R. who are experienced in qualitative interviewing on sensitive topics. Interviews began with a broad question avoiding medicalized language: ‘Would you like to start by talking about when you first noticed a problem?’ We then used a flexible topic guide with prompts (Appendix S1; see Supporting Information) to encourage participants to give their accounts regarding their experiences and perceptions of VLS symptoms, help‐seeking, treatment and management of the condition. The topic guide was developed in partnership with our patient advisory panel. We made field notes to capture additional information and document emerging ideas. Audio‐recordings were transcribed verbatim, checked for accuracy and pseudonymized, with potentially revealing information removed to ensure confidentiality but retain the original meaning.

### Data analysis

Pseudonymized transcripts were organized using QSR NVivo 12. Data collection and analysis was conducted concurrently. Analysis involved a constant comparative method. Two authors (S.A. and S.R.) independently coded and discussed several early transcripts to agree on the coding approach. However, this was not prescriptive given the exploratory grounded theory approach being used. Charmaz^25^ suggests coding data using descriptive labels to summarize ‘what is going on’ in excerpts of data, creating numerous codes per interview, which are then grouped under umbrella nodes. Through this continuous iterative process, categories and themes were developed to build an interpretation.

### Rigour

The authors met regularly to discuss analysis and potential categories/themes. We presented our initial findings to our patient advisory panel, who informed our final thematic structure. One member of our patient panel (S.F.) is a co‐author. Quotations have been provided to support our interpretation.

## Results

### Participants

Seventy‐one women expressed an interest in participating in the study. Forty‐one provided demographic data for purposive sampling. Meaning saturation was achieved after 20 interviews (between November 2020 and May 2021). We felt that we had rich data for our emerging categories. The mean interview duration was 60 (range 45–80) min. Eight were conducted by telephone, and 12 by Microsoft Teams. The remaining individuals who provided demographic data were invited to take part in a separate study looking at quality of life measures, to be reported separately.

Participant characteristics are presented in Table 1. All identified as female, and the majority (80%) were white. We did not receive any interest from trans or nonbinary individuals. The mean age was 53 (range 23–72) years. The mean number of years since diagnosis was 6 (range < 1–23) years. Eleven women reported that their LS was currently under control or being maintained, while nine were experiencing flare‐ups.

**Table 1:** Participant characteristics

Characteristic	*N*
Age (years)	
20–29	2
30–39	2
40–49	3
50–59	4
60–69	7
70–79	2
Ethnic identity	
Asian or Asian British	0
Black or African or Caribbean or Black British	2
Mixed or Multiple Ethnic Group	2
White	16
Type of VLS diagnosis	
Clinical	11
Biopsy	8
Not sure	1
Years since diagnosis	
< 1	3
1–4	8
5–9	5
10–14	2
15–20	1
> 20	1

VLS, vulval lichen sclerosus

### Themes

**Figure 1:**
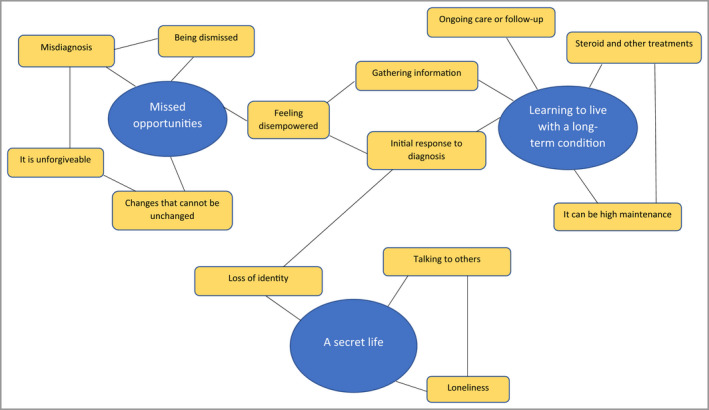
Schematic of themes. [Colour figure can be viewed at wileyonlinelibrary.com]

Three overarching themes with subthemes were developed from the interview data (Figure 1): missed opportunities; learning to live with a long‐term condition; and a secret life.

#### Missed opportunities

Difficulties in getting a timely diagnosis of VLS and/or receiving appropriate information to help understand the condition from healthcare professionals (HCPs) were common. Participants shared their experiences of misdiagnosis, not being examined, being dismissed and a lack of HCP knowledge. These missed opportunities led to anger and disappointment in their VLS journey. Having to ‘fight’ (Paula) for the correct diagnosis and a lack of clear information about the condition and how to manage it left them feeling disempowered and distressed by the potential consequences of these delays on disease progression (Table 2).

**Table 2:** Interview data demonstrating themes of missed opportunities

Theme	Interview data
Misdiagnosis	‘And when I kept going backwards and forwards to the doctor, no one would examine me, no one would, no one was really bothered or interested … I was very raw, and it was really painful just to even have a wee. And she gave me a different treatment for thrush. And it still didn’t get any better, funnily enough.’ (Hazel) ‘I, it was then on my fourth visit I got, I actually got examined. Well, it’s actually, it was the second time I got examined. It wasn’t picked up the first time.’ (Catherine)
Being dismissed	‘I went to a gynaecologist but she was horrible to me … she just dismissed it. So, I think it’s already hard enough to bring it up with your doctor to start with. But when you get dismissed it’s just, it really gets you down sometimes.’ (Madeleine) ‘Well, I just felt like the, the meeting, the appointment was a waste of time. I felt I’d been just dismissed, you know, “You’ve got a touch of Lichen Sclerosus,” is what he put in the letter to the doctor.’ (Fiona) ‘… he was really, really dismissive and on the, on the border of being rude really. Basically said, “Well, why don’t you just go to the chemist and buy your own thrush treatment?” And, and didn’t examine me or, or do anything really. So, at that point I was quite upset and felt abandoned, and nobody cared.’ (Hazel) ‘… the ones that were quite dismissive of me made me feel ten times worse … it’s hard when somebody’s telling you it’s in your head. It just makes you feel like you’re going crazy.’ (Grace)
It is unforgiveable	‘I’m, as I say, I’m just incredulous that it took three gynaecologists, you know, and even then, you know, it was me that put the suggestion there.’ (Yvonne) ‘I was shocked and a bit angry that it had just been put down to being post‐menopausal.’ (Elizabeth)
Changes that cannot be unchanged	‘And now I have got some disfigurement from scarring I am angry about that … I wonder if, if it would have been spotted before … that would have allowed me to then have treatment to prevent the scarring that I now have.’ (Tanya) ‘I was angry that as a woman, we’re always told to check our breasts, but we’re never told to check [our vulvas] … But for me, quite a lot of my anatomy has changed. I’ve got no idea how long I’ve had, had it for. And when I did eventually look with a mirror, I was horrified because it no longer, my, my vulva no longer looks like it should … could we have diagnosed sooner and stopped that happening?’ (Patricia)
Feeling disempowered	‘He just wrote out this prescription for this cream, said, “Use it for two weeks once a day and, and you know, you should be sorted” and that was it.’ (Fiona) ‘And she prescribed Dermovate cream to just, kind of, use as, when I needed it to keep it at bay. And that was more or less the end.’ (Amanda)

##### Misdiagnosis

Those interviewed reported misdiagnoses of menopause, sexually transmitted infections and even helminths. However, most commonly, VLS was incorrectly diagnosed and treated as vulvovaginal candidiasis:

‘It was misdiagnosed as thrush for about 4 years. It was absolutely horrendous because no matter what thrush cream they gave me nothing really helped. And every time they’d do a swab they’d say, “Well, you know, you haven’t got thrush” but they always checked me for thrush.’ (Janice)

Frequently, this diagnosis was made based on the participant’s description of their symptoms alone and very few were actually examined.

When women were diagnosed with VLS, they often framed these instances as chance events, or a piece of good fortune:

‘… by chance I visited the GP [general practitioner] because I’d got some really bad tears. And there was a dermatologist who’d come in that day … they asked if I was okay with an examination. And said, “I think I know what this is, I think you should send this patient for a biopsy.” That was my lucky day.’ (Sandra)

##### Being dismissed

Further opportunities were missed as women described being dismissed by HCPs when they tried to describe their symptoms and feelings, which for some was an enormous challenge given their embarrassment. Seeking empathy and reassurance, they were instead left feeling distressed, upset and abandoned:

‘I remember driving home and crying, it was just such a horrible experience to have to show an intimate area of myself to someone who was quite dismissive and, sort of, derogatory in the way that they spoke to me.’ (Amanda)

Conversely, those with an empathetic HCP described more positive experiences. Their encounters left them feeling reassured and listened to even if their primary HCP was unable to give them a diagnosis but referred them on to a specialist:

‘She made me feel confident that I would be okay, that I was being cared for. I thought, “Well, thank God for that. At least I’m gonna find out what’s up with me.”’ (Patricia)

##### It is unforgiveable

In their search for a diagnosis, some women *were* examined but VLS was not diagnosed. They felt that this was due to lack of knowledge among HCPs but found it difficult to accept:

‘I remember saying “I’m really uncomfortable, I think I’ve got thrush.” She looked and said, “You probably have, try this.” She recommended something like nappy cream which sent me through the roof. That was an opportunity for diagnosis missed. I can’t forgive her for that, to be honest.’ (Sarah)

##### Changes that cannot be unchanged

These missed opportunities for timely diagnosis and management of their VLS led to feelings of concern and anger that the delay had caused damage, describing changes to their anatomy and way of life that could not be undone:

‘She said, “It’s a precancerous skin condition.” And I’m like, “That’s great,” ‘cause I’d been misdiagnosed for 4 years. I’ve lost a lot of structure down there. I think a lot of it went in the first 4 years over not having the correct treatment. Once it’s gone there’s no going back from that.’ (Janice)

##### Feeling disempowered

Interviewees felt that the point of diagnosis was an ideal opportunity to educate and empower them to deal with the condition. Instead, a lack of information about VLS and its management left them feeling disempowered and unsure about how to proceed:

‘No‐one ever gave me any information. Nobody told me to use like a moisturizer or anything at all. That wasn’t discussed.’ (Paula)

#### Learning to live with a long‐term condition

This theme includes participants’ accounts of dealing with and accepting a new life‐long diagnosis, learning how to live with and self‐manage the condition, the treatment involved and thinking about the future (Table 3).

**Table 3:** Interview data demonstrating themes of learning to live with a long‐term condition

Theme	Interview data
Initial response to diagnosis	‘I actually felt very ashamed of myself. I thought it had to be down to me.’ (Catherine) ‘It’s not something that you want to have. I just think I’m a bit unlucky to have it. And I wish I didn’t.’ (Nicola) ‘I would have been 20 years old at that point, thinking, “Oh my god, I’ve got this disease for the rest of my life.” I remember leaving the room in tears and ringing my mum, I was absolutely distraught. I felt quite ashamed and, sort of broken.’ (Grace)
Gathering information	‘There hasn’t been research done, I looked. I was looking through everything and there was nothing.’ (Elizabeth) ‘… looking on the net because there wasn’t any other option.’ (Catherine)
Taking control of my treatment	‘I think you sort of realize things that you can and can’t do … But yeah, it’s, it’s, sort of, trial and error because even with the steroid creams, you know, they don’t always suit some people.’ (Yvonne) ‘Well, I’ve heard some people say twice a week, don’t they but … yeah, I’ve just been using it once a week.’ (Paula)
Ongoing care or follow‐up appointments	‘… the American people, and the Canadian people, they seem to, because they do have, have gynaecologists as a way of life … they, they get monitored. And that’s something that’s totally lacking here …’ (Fiona) ‘It would have been nice of someone to say okay; you’ve got lichen sclerosus, you’re gonna need steroid cream, you’re gonna need to do this but we also recommend that you do this and this. Come back in 3 months’ time and we’ll see how you’re getting on.’ (Janice) ‘I said about the annual check‐up thing and said, “I’ve never been offered that or anything.” She said, “Well, why don’t you come to me in a year and I can have a look? And then we can do the same thing, refer you on if there’s anything that looks concerning.” So, yeah, I left really happy and really reassured.’ (Tanya) ‘If I could find a local dermatologist, I would consider going privately, maybe once every 6 to 12 months, just for that peace of mind.’ (Patricia) ‘It seems like some people get regular check‐ups. I’m not sure whether I need to.’ (Amanda)
It can be high maintenance	‘I seemed to be doing something to myself every time I went to the toilet and having to be really precise with how I looked after my skin. I stopped using toilet paper. I cut up an old fleece blanket into little toilet‐paper‐sized bits. And started using that whenever I had a wee.’ (Hazel) ‘I’ve, I’ve had to change all my underwear. I’ve now bought men’s cotton boxer shorts because they don’t fit me so closely. So, it’s changed my clothing.’ (Elizabeth) ‘… it’s really messy and actually that also interferes with my life as well because I, you know, depending on what I’m going to wear or depending on, you know, plans and stuff, I have to work around when I’m going to put the cream on.’ (Paige)

##### Initial response to diagnosis

Upon diagnosis, several women described how they would never have considered VLS as a possibility because they had ‘never heard of it’ (Sarah, Paige, Hazel, Janice, Tanya) but how suddenly ‘a lot of things made sense’ (Annette). They described mixed emotions including happiness, relief, shame, fear, anger, depression and the shock of learning they had a life‐long condition:

‘I had two thoughts at the time, severe depression at the thought of having this illness for the rest of my life and relief that I’d finally got a diagnosis for all the problems.’ (Carol)

##### Gathering information

To gain some control over their condition, women described doing their own research. They used the internet and online support groups to look for guidelines, research, and to find other people with VLS to talk to, learn from and share experiences with:

‘[The online support group is] my main source of information just because these are women who are going through it … there are things they’ve suggested that I’ve used and it’s helped me a lot.’ (Paige)

##### Taking control of my treatment

Prescription of and advice on how to use steroid treatments varied, often involving an element of trial and error, with differing HCP opinions regarding steroid regimens, leading to confusion and frustration. This conflicting advice led some women to make their own decisions about how often to use their steroid:

‘You should be using it once a day and then another doctor says, “Oh, no, it’s only once a week.” And you say, “Well, what do I do?” But I’ve decided now that I’ll just do maintenance twice a week and if I feel the need for it I’ll do it every day, it’s up to me.’ (Teresa)

However, making these kinds of judgements did not sit comfortably with everyone:

‘It’s very stressful trying to use your own judgement to work out when is enough and when you should stop your steroid.’ (Grace)

While acknowledging the importance and benefit of their steroid treatments, some women also advocated the use of adjunct treatments, e.g. emollients or moisturizers. They learnt about these from either HCPs with more in‐depth knowledge of the condition or, more often, from support groups. Some felt that information about these other products was part of the missing information that should have been included in a ‘package of care’ (Amanda) given at the point of diagnosis:

‘Having joined a Facebook group and gone through hours of posts I went back to the GP and asked if I could have Hydromol to moisturize and to wash with.’ (Patricia)

##### Ongoing care or follow‐up appointments

Ongoing check‐ups were considered very important. However, as with other aspects of the treatment for VLS, there was variation in follow‐up care: some women were having regular reviews with a National Health Service or private consultant or their GP, some had never had a check‐up, and some were unsure if they should be having regular follow‐up. Those having regular follow‐up felt supported and reassured:

‘They examine me and make record of any changes and I can talk over any concerns with them and they keep a close eye on me which is wonderful.’ (Janice)

##### It can be high maintenance

The impact of VLS on everyday life was summed up as ‘high maintenance’ (Madeleine). This encompassed multiple things including the time and work involved in applying treatments, changes to clothing, toileting, sleep, sex and intimacy, working life, changing hobbies or activities, planning ahead and the need for a routine:

‘I’ve got used to the routine now because there’s extra jobs to do. It’s not spending a penny anymore, it’s, like, spending 10 pence because it takes such a long time. I’ve got to find a proper toilet and probably go in the disabled persons’ toilet while I do all my creaming and things.’ (Teresa)

#### A secret life

Given the lack of widespread knowledge and understanding about VLS, for some women, living with the condition was like living a secret life (Table 4).

**Table 4:** Interview data demonstrating themes of a secret life

Theme	Interview data
Talking to others	‘My second husband is absolutely amazing. I know that if I ever have any problems or issues I can talk to him, he’s fully supportive of me.’ (Janice) ‘… it’s not something I would talk to people about. I just think it’s not a very nice thing to have and, and, and that it’s not something you bandy about in conversation.’ (Nicola) ‘We’re really, really close but couldn’t do it. I didn’t want to explain the awkwardness and make her feel awkward. So, I’ve just got to the point where I thought, “Do you know what? I’m not gonna go there.”’ (Sandra) ‘And I couldn’t, I, I can’t bear the questions … you know, what conclusions people are gonna come to.’ (Annette) ‘I still haven’t told my daughters, but I intend to. But it’s taken me a long time to get to this place. And I will tell them, because we have granddaughters as well now.’ (Catherine)
Loneliness	‘… you’re having a bad week and you’re trying everything and it’s not working, I mean, if you can’t talk to anyone or ask anybody about it, you feel a bit … it’s a lonely disease anyway.’ (Sandra) ‘… it’s a phenomena, isn’t it, that when women get together, they help each other, and Facebook has been unbelievable in that respect.’ (Carol) ‘LS is about loneliness. Dealing with it … talking about it but dealing with it alone. That is what LS is about.’ (Camille) ‘Thankfully I found a Facebook group for women in the UK. So that was really comforting to hear their stories … to be able to talk freely.’ (Paige)
Loss of identity	‘Some days it does bother me and think I’m, I’m a less of a woman ‘cause I can’t do what I want to. But then I do have some days, I, I have good days I try and make up for it.’ (Janice) ‘I mean women’s bodies change obviously as we get older anyway, but it doesn’t make you feel very attractive when you know that something’s not quite right down there.’ (Yvonne) ‘The fact that you lose architecture, all of that which is part of how you define yourself as a woman, suddenly begins to be eroded.’ (Elizabeth) ‘LS rob[s] us women of lots of things … your dignity … it, it makes you hate part of your body’. (Camille)

##### Talking to others

Often, those in relationships described how supportive, and understanding their current partners were. However, many women had told almost no one about their condition, instead making dramatic changes to their lives in secret:

‘I know there are other people like me who have changed how their life is because of it. And we do it secretly ‘cause you don’t tell anyone. It’s not something you talk about; I don’t discuss this with anyone.’ (Sandra)

Talking about it was made more difficult because some felt ashamed about their diagnosis, worrying about how others would respond, which was compounded by a lack of knowledge about VLS and female genitalia in general:

‘So, I’ve only mentioned it to two [friends] and in both cases I felt the need to say it’s not sexually transmitted. And I’m not normally like this, it’s only with the LS.’ (Sarah)

##### Loneliness

Given people’s lack of awareness, and the shame and stigma precluding them from sharing their experiences with others, VLS was described as ‘quite isolating really’ (Fiona) and a ‘lonely condition’ (Madeleine).

Support groups were considered invaluable in tackling the loneliness associated with the condition. Women were thankful for these groups, learning from others’ experiences diminished feelings of shame and isolation offering comfort in that ‘there are people in the same boat as I’m in’ (Janice):

‘I have since joined many groups and they’ve been so helpful. It just makes such a difference to know you’re not on your own.’ (Annette)

However, support groups were sometimes considered ‘a double‐edged sword’ (Tanya) because comparison with others’ suffering left some women distressed and fearful about their own VLS:

‘They were fuelling my anxiety because I was hearing too many sad stories. They served a purpose; they gave me a few ideas. But I stopped looking at them and I didn’t wanna hear everybody’s heartache stories.’ (Hazel)

##### Loss of identity

As well as keeping their VLS a secret, some women described a loss of identity, sense of self or femininity, and their feelings towards their body changed:

‘I just said to my husband, “I completely feel not like a woman.” I feel like I’ve got this invisibility cloak.’ (Sandra)

## Discussion

This was the first UK study to specifically explore the experiences of women with VLS from their own perspectives. We used in‐depth interviews to enable women to tell their stories, and social constructionist grounded theory analysis to develop an interpretation grounded in their accounts.^25^ Our findings build on and extend previous research,^17^, ^20^, ^22^, ^23^ highlighting issues of delayed diagnosis, poor HCP attitudes, inadequate information provision to support self‐management and the ongoing stigma and secrecy surrounding vulval conditions.

A decade on from the research by Wehbe‐Alamah *et al*.,^17^ our participants’ stories demonstrate that women continue to struggle to obtain a timely and accurate diagnosis of VLS. Being dismissed is a theme which echoes throughout the body of existing qualitative research on women’s experiences of VLS.^20^, ^23^ This results in women having to emotionally prepare for attending or even phoning their doctors’ surgery, if they anticipate resistance to accessing diagnosis or their treatment.

In our study and others,^17^, ^20^, ^23^ delayed diagnosis was blamed on a lack of HCP knowledge about VLS and, therefore, delays in appropriate onward referrals. The women in our study felt that education about VLS for HCPs should be a priority to improve the experience of diagnosis. HCPs should always consider VLS as a possible diagnosis in patients presenting on multiple occasions with vulval symptoms, should examine and know what to look for, or know who to refer patients on to if they are uncertain. Positive experiences with HCPs arose when their concerns were taken seriously and when they were given more support and information about their condition.

Having been diagnosed, women were eager to self‐manage their condition but were not always provided with adequate information or follow‐up support to do this. It was felt that a ‘package of care’ at the point of diagnosis, including all the relevant information about the condition, would have been really helpful. Instead, and similar to the findings of Rivera *et al*.,^23^ women sought out information and engaged in ‘self‐advocacy’ to gain appropriate treatment, make decisions about their treatment and even educate HCPs in the process. However, this was experienced as a source of stress, and not expressed in a positive light, challenging to some extent Rivera *et al*.’s interpretation of their survey‐based data.^23^ The notion of the ‘expert patient’ who advocates for themselves has been critiqued for being individualistic and not accounting for structural inequalities and the power dynamics of the patient–doctor relationship.^26^

Similar to the findings by Wehbe‐Alamah *et al*.,^17^ the women in our study sought advice and peer support online. However, our work highlights how membership in these groups was not straightforwardly positive. They were grateful for relevant information about VLS but found witnessing other women’s suffering anxiety‐inducing or distressing.

The British Association of Dermatologists guidelines for the management of LS^2^ recommends follow‐up in secondary care every 6–12 months until treatment success is achieved and suggests a yearly check in primary care. Others have expressed concern about inconsistency in follow‐up because of the heightened (although small) risk of vulval cancer.^27^ Our data suggest that women across the UK are not routinely or consistently offered follow‐up appointments but feel that this is crucial to help women understand if/how their disease is progressing and for provision of information and support. Regular, routine follow‐up would be consistent with recognizing that VLS is a disease that is chronic and has a major impact on quality of life,^12^, ^13^ for which women need support to manage.

Our findings also indicate that educating the general public about VLS and female anatomy is crucial to address the stigma associated with women’s vulval health. The general population’s knowledge about vulval/genital anatomy has been shown to be poor.^28^ Other research on LS has also identified this to be an issue.^17^, ^20^ Addressing this may require a public health approach to change the culture which causes some women to live secretly with VLS.

Regarding strengths and limitations, this was the first UK study to use qualitative interviews with a sample of women with VLS to explore their experiences from their own perspectives. The use of established theoretical frameworks in material development and data analysis, and the Standards for Reporting Qualitative Research^29^ to report the findings, provides a strong evidence‐based foundation to our findings.

Recruiting from social media and online support groups meant that our findings might not be transferable to women who do not engage with these resources. Women seeking online support may be particularly anxious about their disease. This group may also have had more negative encounters with HCPs which increased their need for support. The use of interviews may have excluded some people who would prefer to give their account anonymously such as via a survey or written response. However, the option of telephone interviews offered women some sense of anonymity from the researcher. The small numbers of black, Asian and other minority ethnic participants in our study made it challenging to draw particular conclusions about their experiences, and future research could seek to include only these participants. Further research is also needed to understand the experiences of trans and nonbinary people with VLS.

In conclusion, this study adds to the growing body of evidence that the needs of women with symptoms of VLS are not being met sufficiently by the healthcare system. The wider issues of societal attitudes towards women’s health and especially women’s genital health require addressing with a sociocultural and public health approach, but meanwhile HCPs could do more to improve women’s experiences of seeking and receiving care for their VLS. HCPs’ awareness of the spectrum and range of women’s genital health disorders needs improvement and they should take the greatest of care to be as sensitive, understanding and supportive as possible when women seek care for vulval symptoms or to help manage their VLS. A consistent and evidence‐based package of care should be provided to all women with VLS, so that they feel well supported and informed about their condition.

## Funding

Economic and Social Research Council (ESRC) Ref: ES/T005939/1

## Conflicts of interest

The authors declare no conflicts of interest.

## Ethics statement

This study was approved by the University of Warwick Biomedical and Scientific Research Ethics Committee (BSREC), Ethical Application Reference: BSREC 02/20‐21.

## Supporting information


**Appendix S1** Interview schedule: living with vulval lichen sclerosus.

## Data Availability

The data that support the findings of this study are available on request from the corresponding author. The data are not publicly available due to privacy or ethical restrictions.
