# Supplementary material for: Living with vulval lichen sclerosus: a qualitative interview study
Source: Br J Dermatol. 2022 Aug 22;187(6):909–18. doi: 10.1111/bjd.21777 (PMC10087446; doi:10.1111/bjd.21777)
Supplement: Supplementary file 1 — Appendix S1 Interview schedule: living with vulval lichen sclerosus. [file BJD-187-909-s001.docx]

**Supplementary information**

**Appendix S1: Interview Schedule: Living with Vulval Lichen Sclerosus (LS)**

This qualitative study will involve individual interviews. Please ensure all participants have read the Patient Information Leaflet (PIL) and signed a consent form before their interview.

***Reminder – Purpose of interviews***

1. To explore participants’ experiences of living with vulval LS.
2. To explore the impact of the condition on their identity
3. To explore the participants’ experiences of seeking/accessing medical care and attitudes towards LS treatments
4. To explore questions relevant for clinical research into LS

**Before switching on the audio-recorder**

*Background information – why this interview*

“This interview is to help us understand your experience of living with LS.”

*Confidentiality*

“Before we start talking, I would like to assure you that everything that you say will remain confidential. Your doctor, nurse or any other health professional will not see or hear any of the information that is shared here. If we do use anything that you have said, such as in a report or journal article, it will be made anonymous so that you cannot be identified.”

*Any questions at this stage?*

“Do you have any questions before we switch on the recorder and start?”

**Switch on the audio-recorder**

Interviewees will be encouraged to speak openly and freely about their experience of living with LS.

Indicative questions and prompts are given below (these questions are flexible and not all questions will be appropriate or asked of all participants).

**Open with:**

**‘Would you like to start by talking about when you first noticed a problem?’**

| **Topic** | **Prompts** – *as appropriate follow up with probes e.g. ‘why/not’, ‘could you expand/tell me a bit more on that?’, ‘what was/is that like for you?’* |
| --- | --- |
| Everyday life | In what ways does LS affect your day-to-day life? |
| Health care seeking | Can you talk about your experiences with healthcare professionals (like doctors, nurses etc.) in relation to your LS?  Where did you seek help? (if needed prompt e.g. GP, sexual health centre)  What prompted you to go and see that healthcare professional? |
| Talking about LS | Do you find it easy to talk about LS with other people (e.g. partner, friends, family)  (if not) What is it that makes talking so difficult? Does it get easier over time?  How does that make you feel? |
| Information | Where did you find out about LS?  Where do you get your information about LS?  What sort of information has been helpful to you? For example, information from a doctor, on the NHS site, from support groups, other women? |
| Management | Did/do you use steroid ointment? What is that like?  What else do you do to help prevent a flare up?  What else do you do to relieve symptoms? |
| Sex/relationships *(if they are willing to speak about it)* | Has LS affected your relationships? Sex life? How have you managed that? If yes, how does that make you feel? |
| Identity | Has LS affected the things you used to do - for example sport, hobbies, socialising, work?  Do you feel that LS has affected how you feel about yourself? In what ways?  Do you feel that LS has affected your sense of femininity?  Has LS affected your confidence? |
| Outcomes | What in particular about living with LS do you think researchers should be trying to improve?  *Allow woman to give any spontaneous answers, or if needed provide examples*: particular symptoms, preventing vulval cancer, improving confidence in self-managing the condition. |
| Research | If there was a study comparing ‘real’ steroid ointment with ‘fake’ placebo cream, would you be willing to take part in it? In the study you wouldn’t know if you had the real or the placebo ointment.  What if it was a trial of another type of treatment?  Is there anything else that you think should be being researched? |
| Towards end | Is there anything that has helped you/what has helped you most when coping with LS? |
| End | Is there anything else we haven’t talked about that you would like to say? |
